# Instrument shank-assisted ovariohysterectomy: a randomized clinical trial of surgical and pain alleviation efficiency of a single-person modified technique

**DOI:** 10.3389/fvets.2023.1210089

**Published:** 2023-10-17

**Authors:** Navid Ziaei Darounkolaei, Seyed Mohamad Sadegh Mousavi Kiasary, Amirhoushang Behzadi, Niki Nabavi Mosavi, Shima Mahmoodi Ferdowsi

**Affiliations:** ^1^Babol Branch, Department of Surgery and Radiology, Faculty of Veterinary Medicine, Islamic Azad University, Babol, Iran; ^2^Health Policy Research Center, Institute of Health, Shiraz University of Medical Sciences, Shiraz, Iran; ^3^Nano Bio Electronic Devices Lab, Cancer Electronics Research Group, School of Electrical and Computer Engineering, College of Engineering, University of Tehran, Tehran, Iran; ^4^Babol Branch, Faculty of Veterinary Medicine, Islamic Azad University, Babol, Iran

**Keywords:** instrument shank-assisted, ovariohysterectomy, pain score, deep-chest, dog, OHE

## Abstract

**Objectives:**

To evaluate a modified ovariohysterectomy (OHE) technique performed by a single person and compare it with the conventional method based on time efficiency, trauma, and postoperative pain.

**Methods:**

In a prospective, randomized, experimental study, 18 healthy, large, deep-chested, mixed-breed intact female dogs were randomly allocated to conventional (*n* = 9) and instrument shank-assisted (*n* = 9) groups. On the basis of video recordings, the various surgical step durations were analyzed: total surgery time (TST), pedicle intervention time (PIT), suspensory release time (SRT), shanking time (ShT), clamping time (ClpT), ligating time (LigT), and closure time (CT). The Glasgow composite pain scale short-form (GCMPS-SF), university of Melbourne pain scale (UMPS), and Visual Analogue Scales (VAS) were used to measure pain. C-reactive protein (CRP) fluctuation was also investigated. These evaluations were completed before and 6, 24, 48, and 72 h postoperatively.

**Results:**

Instrument shank-assisted OHE was less time-consuming than conventional OHE (*p* = 0.005), improved PIT by 30.7% (6.44 min for both pedicles, *p* = 0.014), and correlated strongly with TST (*ρ* = 0.862, *p* = 0.003 and ρ = 0.955, *p* = 0.000, respectively). The two method’s surgical step durations were also TST = 47.40 ± 9.9 vs. 34.70 ± 6.7 min, PIT = 20.96 ± 5.78 vs. 14.52 ± 3.73 min, SRT = 78.97 ± 69.10 vs. ShT = 20.39 ± 8.18 s (*p* = 0.035), ClpT = 50.66 ± 45.04 vs. 63.55 ± 37.15 s (*p* = 0.662), LigT = 12.82 ± 3.37 vs. 8.02 ± 3.11 min (*p* = 0.005), and CT = 16.40 ± 4.5 vs. 11.60 ± 2.5 min (*p* = 0.013), respectively. While both techniques inflicted pain on the animals, the novel approach resulted in a reduction of pain at T6 (GCMPS-SF, *p* = 0.015 and VAS, *p* = 0.002), T24 (UMPS, *p* = 0.003), and T48 (GCMPS-SF, *p* = 0.015 and UMPS, *p* = 0.050). Both methods exhibited a peak in CRP level after 24 h, which subsequently returned to baseline after 48 h. However, the shank-assisted method demonstrated a significantly lower reduction in CRP level at the 48-h compared to the other group (*p* = 0.032).

**Conclusion:**

Instrument shank-assisted technique permitted ovarian removal without an assistant, less damage to animals and reducing its time when compared to a conventional technique, and resulting in an alternative that causes less surgical stress and fatigue. Further research with a larger population size is required to determine the serum CRP levels as an alternative pain biomarker.

## 1. Introduction

Elective ovariohysterectomy (OHE) is one of the most common surgeries done on dogs and cats (1). However, there are still problems with this technique, despite how common it is. Prior to the start of the operation, inexperienced graduates are anxious about how to do OHE on their own, and after the procedure is finished, they are concerned with the reliability of ligatures. As they consider how to do this procedure at an acceptable speed, their anxiety will increase. When a surgeon acquires experience, new issues develop, such as how to properly perform a high-risk, quick surgery with several implications, such as hemorrhage (2, 3), surgical trauma, organ manipulation, inflammation (4, 5), surgical stress (6), wound healing, and acute pain. The frequency of ovarian remnant syndrome was increasing mostly among young surgeons because of a concern about vascular rupture while breaking the suspensory ligament and exposing the ovaries inadequately, particularly in patients with obesity or other comorbidities (7–9). According to Berzon’s study, 1% of bitches experienced recurrent estrus following OHE by fourth-year veterinary students (2). However, the overall frequency of complications by final-year veterinary students was found to be as high as 29 (20.6%) out of 141 bitches, with 1 (5%) out of 20 experiencing post-surgical pseudopregnancy (7). As experience grows, less consideration is given to this problem. To achieve the aforementioned results, the surgeon may be skilled in rupturing the suspensory ligament to expose the ovaries and make their pedicle accessible for ligature placement (10). The success of the operation hinges on the surgeon’s willingness to pull, compress, strumming, tear, or sever the suspensory ligament (11), which is accompanied by extensive tissue damage. Drastic tissue damage and the time-consuming nature of its execution have made it the leader in painful surgeries in veterinary medicine, particularly for inexperienced surgeons. The experts, anesthesiologists, surgeons, and experienced researchers have adopted OHE as the acute surgical pain model because of the intensity of the pain caused by an experienced surgeon’s OHE (12–14). Acute postoperative pain has long been a problem for surgeons. Inadequate management of postoperative pain can result in a number of undesirable outcomes, including (1) physiological changes comprising tachycardia, hypertension (due to peripheral vasoconstriction, increased myocardial contractility, and systemic vascular resistance), cardiac arrhythmias, tachypnea, superficial respiratory pattern, pale mucous membranes, mydriasis, sialorrhea, and hyperglycemia (2), behavioral changes comprising vocalization (such as cries, whimpers, and growls), looking and licking the affected area, alteration of the facial expression (submissive attitude), self-mutilation, muscle stiffness or weakness, restlessness and anxiety, apathy and inactivity, aggression, fear, and depression, stereotypes, anorexia or hyporexia, reduction of grooming, prayer posture, sleep disorders, and (3) changes in biochemical parameters by the decrease of PaO_2_, PaCO_2_, HCO_3_, and an increase of H^+^, cortisol, lactate, and glucose (15–20), prolonging the recovery of patients (17, 21, 22). Despite these hazards, OHE is considered to be a very straightforward procedure, and numerous dog owners visit a veterinary clinic every day to have their pets spayed. Hence, the incidence of problems justifies the adoption of procedures, such as instrument shank-assisted OHE, as well as the many theories pertaining to surgical duration, pain, and trauma.

The length of surgery is a primary factor of the severity of postoperative issues, and it is inversely related to the surgeon’s skills and expertise (13). Skill is considered in two fields: non-technical skills (e.g., knowledge, situational awareness, decision-making, conscientiousness, intraoperative communication, teamwork, and leadership) (23–25) and technical skills (psychomotor actions). The latter would be gained and empowered via an educational program known as Objective Structured Assessment of Technical Skills (OSATS), which has been thoroughly introduced and verified (26, 27). Besides these abilities, some surgical procedures, such as minimally invasive procedures, are time-consuming and instrument-dependent (28, 29), which may not be an option for some animals. Naturally, each operation consists of a succession of procedures with varying durations, since some are simpler than others, such as entering the abdominal cavity through the linea alba, while others, such as the anatomical access to the ovaries, provide challenges. Understanding the elements that determine the duration of surgical steps as well as the total duration makes it simpler and more objective to estimate its sufficiency and leads to a more trustworthy conclusion.

Animal pain is hard to judge because it depends on many things, such as the amount of pain, the type of injury, and the animal’s own characteristics. As a result of the intricate nature of pain perception, several multidimensional questionnaires for qualitative pain assessment and validated behavioral scales have been developed to assess pain intensity in dogs (30, 31). Each of these methodologies assigns a different number of points to certain animal behavioral changes. The sum of the points indicates the observed pain level of the animal. Commonly used pain scales include the Glasgow composite measure pain scale (GCMPS-SF), the University of Melbourne pain scale (UMPS), and the Visual Analogue Scale (VAS), with corresponding ranges of 0–28, 0–24, and 0–10. Each of these solutions seems capable of filling some of the gaps left by the others, since they possess almost separate criteria with little overlap (32).

It is considered that the pain will always correspond to specific parameters that changed when the discomfort began or emerged. Clinical studies may describe a vast array of biomarker variations. Some parameters represent a range of events, but others may be directly triggered by the existing pain. The inflammatory response to surgical trauma or stress (33) activates the hypothalamus, causing it to release corticotropin-releasing hormone and arginine vasopressin, both of which stimulate anterior pituitary adrenocorticotropic hormone production, which in turn stimulates cortisol secretion by the adrenal cortex (19). Cortisol levels vary based on the severity or grade of surgery. The surgical interventions were categorized based on the modified Johns Hopkins surgical criteria, which delineate three levels of invasiveness: grade I, indicating minimally invasive procedures; grade II, indicating moderately invasive procedures; and grade III, indicating highly invasive procedures (34). When comparing grade 2 and grade 3 operations to grade 1, these differences may be identified, but they cannot be separated. Cortisol is a commonly utilized measurement for assessing stress levels and has demonstrated efficacy in evaluating intraoperative noxious stimuli. However, its sensitivity may be inadequate for capturing the variations that arise from repeated intraoperative noxious stimuli in a single animal (35). Cortisol levels seem to fluctuate with age, gender, disease, and the degree of surgical or anesthetic invasiveness. As a result, based on the research conducted so far, it is difficult to determine which is the primary cause of the alterations (36). Glucose is another biochemical parameter that surgery affects, and its clinical monitoring appears straightforward. Growth hormone (somatotrophin) levels increase in response to surgery and trauma; their release from the anterior pituitary is promoted by hypothalamic growth hormone-releasing factor (37, 38), which has an anti-insulin effect by inhibiting glucose uptake and utilization by cells. However, glucose utilization by cells is limited during surgery due to high cortisol levels (39). As a consequence, blood glucose levels rise. Furthermore, cortisol and catecholamines promote glucose production. In addition, a hyperglycemic response may result from a drop in insulin concentration during induction of anesthesia and during surgery, resulting in insulin secretion failure. Ultimately, the surgical invasion causes an increase in blood glucose content (40). Regardless of the causes of elevated glucose levels, the amount of rise in simple operations is negligible (19). Immunological mediators such as cytokines or interleukins (ILs) such as IL-1, IL-6, and tumor necrosis factor-alpha (TNF-α) mediate the rapid activation of the immune system following surgery. The presence of IL-6 depends on the extent of the surgical tissue damage (20). Despite the fact that the plasma level of IL-6 molecules with a short half-life increases within 30–60 min and becomes substantial after 2–4 h with quick returns to baseline, the maximum level may be attained 24 h after major operations, which may be prolonged 48–72 h postoperatively (19, 41). IL-6 stimulates the release of proteins, especially C-reactive protein (CRP), from the liver to commence the “acute phase response,” which comprises a variety of changes (19, 42). Based on a comparable study design (42), the postoperative CRP concentration increased more slowly and reached its peak after 48 h. After that, it went down at a slower rate, with a mean half-life of 62 h compared to 15 h for IL-6 (43). This might make it a valuable and accurate marker for regular diagnostics of systemic inflammation in dogs (44, 45) and a predictor of surgical trauma severity (46).

Therefore, one of the most difficult things for surgeons to do is choose a technique that will cause the least amount of damage, cause the least amount of pain after the surgery, and take the least amount of time. These are a trio of the key challenges for surgeons. The development or modification of minimally invasive techniques has only been able to improve the first two of these aspects, but with significant limitations (47). The aim of this study is to compare a modified OHE procedure that only needs one person to do it with the standard procedure in terms of time, trauma, and pain after the surgery. This will help researchers come up with a way to reduce the length of surgery, the amount of trauma, and immediate postoperative pain.

## 2. Materials and methods

### 2.1. Animals and study design

This research was authorized by the Iranian biomedical research ethics committee [IR.IAU.BABOL.REC.1399.004 (48), IR.IAU.BABOL.REC.1399.015 (49) and IR.IAU.BABOL.REC.1399.093 (50)] and conducted at the Babol branch of Azad University.

In a randomized controlled trial, 18 healthy, large, deep-chested intact female mixed-breed dogs were included. Animals were divided into two equal groups randomly by coin flipping, using a sterile suture sachet (51–53) (NZD) after inducing anesthesia and draping the surgical area. The sample size was evaluated using the software GPower 3.1.9.7. The presence of 9 dogs in each group resulted in a power of 0.9 (Power = 1 − β = 0.9) for TST with effect size *d* > 1.50 at a significance level of = 0.05.

Shelter dogs with ASA I (the American Society of Anesthesiologists) physical status enrolled in the study (54). The physical exam checked the patient’s heart rate (HR), breathing rate (RR), and rectal temperature (RT). It also checked for internal and external parasites, did a complete blood count, and looked at the Hb, PCV, CRP, and glucose levels in the blood. The study was conducted on bitches in diestrus, based on vaginal smear cytology. Animals with a body condition score between 4 and 6 out of 9 were chosen. Animals under 1 year old, in estrus, pregnant, or lactating, with a weak or no response to painful stimuli (a needlestick in the lower abdomen), with a history of physical or behavioral issues, or with abnormal vaginal secretions were excluded.

Each dog scheduled for surgery on a particular day spent 3 days before and 3 days after the surgery in a separate cage with free access to food and water. Ten days after surgery, the sutures were removed.

### 2.2. Anesthesia

The animal’s resting vital parameters (HR, RR, and RT) were recorded before anesthesia. Anesthesia and analgesia were provided by acepromazine (10 mg mL^−1^, Neurotranq; Alfasan, Woerden, Holland), midazolam (5 mg mL^−1^, Midazolam; Caspian, Rasht, Iran), pethidine (100 mg 2 mL^−1^, Petholan; Adeka, İstanbul, Turkey), medetomidine (1,000 mcg mL^−1^, Dorbene Vet; Syva, León, Spain) and ketamine (50 mg mL^−1^, Ketamine HCl Inj.; Rotexmedica GmbH, Trittau, Germany), and ketorolac (30 mg mL^−1^, Ketorolac; Alborz Darou, Tehran, Iran). A 19G catheter was placed aseptically in the cephalic vein for a given lactated Ringer’s solution (250 mL, lactated Ringer’s solution; Shahid Ghazi, Tabriz, Iran) at a rate of 5 mL kg^−1^ h^−1^.

The animals were premedicated by acepromazine at 0.02 mg kg^−1^, midazolam at 0.5 mg kg^−1^, meperidine at 2 mg kg^−1^, medetomidine at 20 mcg kg^−1^, and ketamine at 4 mg kg^−1^ IM. They received ketorolac at 1 mg kg^−1^ immediately before surgical asepsis. The maintenance of anesthesia was achieved by administering a consistent anesthetic mixture (ketamine at 4 mg kg^−1^ and midazolam at 0.27 mg kg^−1^) at a variable rate of 0.2–0.5 mg kg^−1^, depending on the ketamine levels present in the mixture. The administration of the anesthetic was monitored through the use of several parameters, including SpO2, ECG, non-invasive blood pressure measured through a blood pressure cuff (size #4) placed proximal to the carpus over the radial artery at five-minute intervals, and respiration. The surgical procedure and manipulation were also taken into account during the administration of anesthesia. The Pm-7000vet, manufactured by Wuhan Zoncare Bio-medical Electronics Co., Ltd., was used to monitor animals. The animals’ cardiorespiratory parameters were monitored until they demonstrated full recovery.

A nociceptive response was defined as a 20% or more rise in heart rate over the base rate, accompanied with an increase in breathing frequency and blood pressure proportionate to a painful surgical procedure (20, 55, 56). Ketamine at 0.5 mg kg^−1^ was used for rescue analgesia during surgery. The timeline details of measures were provided in Table 1.

**Table 1:** The measures carried out during the conventional (*n* = 9) and Instrument Shank-assisted ovariohysterectomy (*n* = 9).

Steps	Time (min)	Medicines/Dose/Application	Rout/Location/Area
Anesthesia, Premedication^1st^	0 (Start)	Acepromazine (10 mg mL^−1^), 0.02 mg kg^−1^	IM
		Midazolam (5 mg mL^−1^), 0.5 mg kg^−1^	
		Meperidine (50 mg mL^−1^), 2 mg kg^−1^	
Vital parameters monitor			
Electrocardiograph	1–2 min after laying down	Lead II	On the elbows and knees
Blood pressure		Neo #4	On the carpus over radial artery
Pulse oximeter		Veterinary SPO2 transducer	On the ear until induction, then on the tongue
IV catheterization	10	19G	Cephalic vein
Preventive antibiotic		Cefazolin (1 g vial^−1^), 22 mg kg^−1^	IV
Fluid therapy		Lactated Ringer’s solution, 5 mL kg^−1^ h^−1^	IV
Hair clip		No 40	Mid-chest to mid-thigh
Anesthesia, Premedication^2nd^	15	Medetomidine (1,000 mcg mL^−1^), 20 mcg kg^−1^	IM
		Ketorolac (30 mg mL^−1^), 1 mg kg^−1^	SC
Aseptic preparation	17	Povidone Iodine scrub (7.5%) and 70° ethyl alcohol, three times consecutively; then Povidone Iodine solution (10%) was applied	
Anesthesia, Induction	20–25	Ketamine (50 mg mL^−1^), 4 mg kg^−1^	IV, Anesthetic mixture
		Midazolam (5 mg mL^−1^), 0.27 mg kg^−1^	
Endotracheal intubation	Immediately after induction	A maximum size based on the rough estimation using the √ (Body weight×5)	Intraoral, mid-trachea
Anesthesia, Maintenance	As needed	Induction anesthetic mixture, 0.2–0.5 mg kg^−1^ based on the ketamine	IV, Anesthetic mixture
Intraoperative rescue analgesia	As needed	Ketamine, 0.5 mg kg^−1^	during surgery based on HR and RR and surgical manipulation
End of surgery (the last skin suture)			
Conventional Instrument Shank-assisted	67–72 55–60		
Postoperative antibiotic	Every 8 h	Cefazolin (1 g vial^−1^), 22 mg kg^−1^	IM, for 3 days
Postoperative rescue analgesia	Every day	Ketoprofen, 2 mg kg^−1^	IM, as needed, at cefazolin injection time evaluation as needed

IM, intramuscular; IV, intravenous; SC, subcutaneous; HR, heart rate; RR, respiratory rate.1st: administration of the first part of pre-anesthetic drugs, which included Acepromazine, Midazolam, and Meperidine; 2nd: Administration of the second part of pre-anesthetic drugs which included Medetomidine and Ketorolac.

### 2.3. Surgery

#### 2.3.1. The surgeon and surgical team

Two months after graduation, a female doctor of veterinary medicine (DVM) with minimum experience (according to the veterinary training course) in the conventional approach and no expertise with the new methodology has been selected as the surgeon (NNM). The selected surgeon and surgical team underwent a one-week training course for each surgery 10 days prior to the start of the study. During the training course, one surgery was done on each technique by the advisor, and then the techniques were randomly performed on 12 dogs (6 dogs each technique) by the surgeon conducting the study. The random approach has been a coin toss (51–53); thus, the first-day method was determined by tossing a coin, and the next day the opposite technique must be followed. The study surgeries were performed by the same team under the supervision of the dissertation adviser (NZD).

#### 2.3.2. Aseptic surgical preparation

In dorsal recumbency, the ventral abdomen was aseptically prepped after hair removal from mid-chest to the end of the pelvic symphysis and the inner thigh. During pre-surgical aseptic preparation, the skin was alternately scrubbed three times with 7.5% povidone-iodine and 70% ethyl alcohol. After the final povidone-iodine scrub is complete, a 10% povidone-iodine solution is applied to the surgical field (1).

#### 2.3.3. Approach and incision length

A ventral midline celiotomy was performed immediately caudal to the umbilicus and extending one-third of the way to the pubic rim (11) for both methods, in order to achieve the same incision length (57, 58).

#### 2.3.4. Surgical methods

##### 2.3.4.1. Conventional (triple hemostatic) OHE

In the control group, the triple-clamp OHE (1) was done after entrance into the abdominal cavity and control of the uterine horn without a spay hook. During this method, the surgeon’s dominant index finger is used to grab the left horn of the uterus. For organ manipulation, rat-toothed Crile forceps secured to the proper ligament were utilized. The suspensory ligament was strummed and released manually in the caudomedial direction. After creating a mesovarium window, two simple ligatures were placed on top of one another in the first clamp crush near the kidney. One transfixation ligature was then tightened in lieu of the middle clamp near the ovary [Polydioxanone (PDS II), 2–0]. The pedicle was transected and inspected for hemorrhage. The same techniques were then conducted on the contralateral pedicle. Separately, the cervix and uterine arteries were ligated (PDS II 2–0). Linea alba (PDS II 0), subcutaneous tissue (PDS II 2–0), and skin (monofilament Polyamide 0) were routinely closed. A stent bandage was then placed over the suture line.

##### 2.3.4.2. Modified instrument shank-assisted OHE

**Figure 1:**
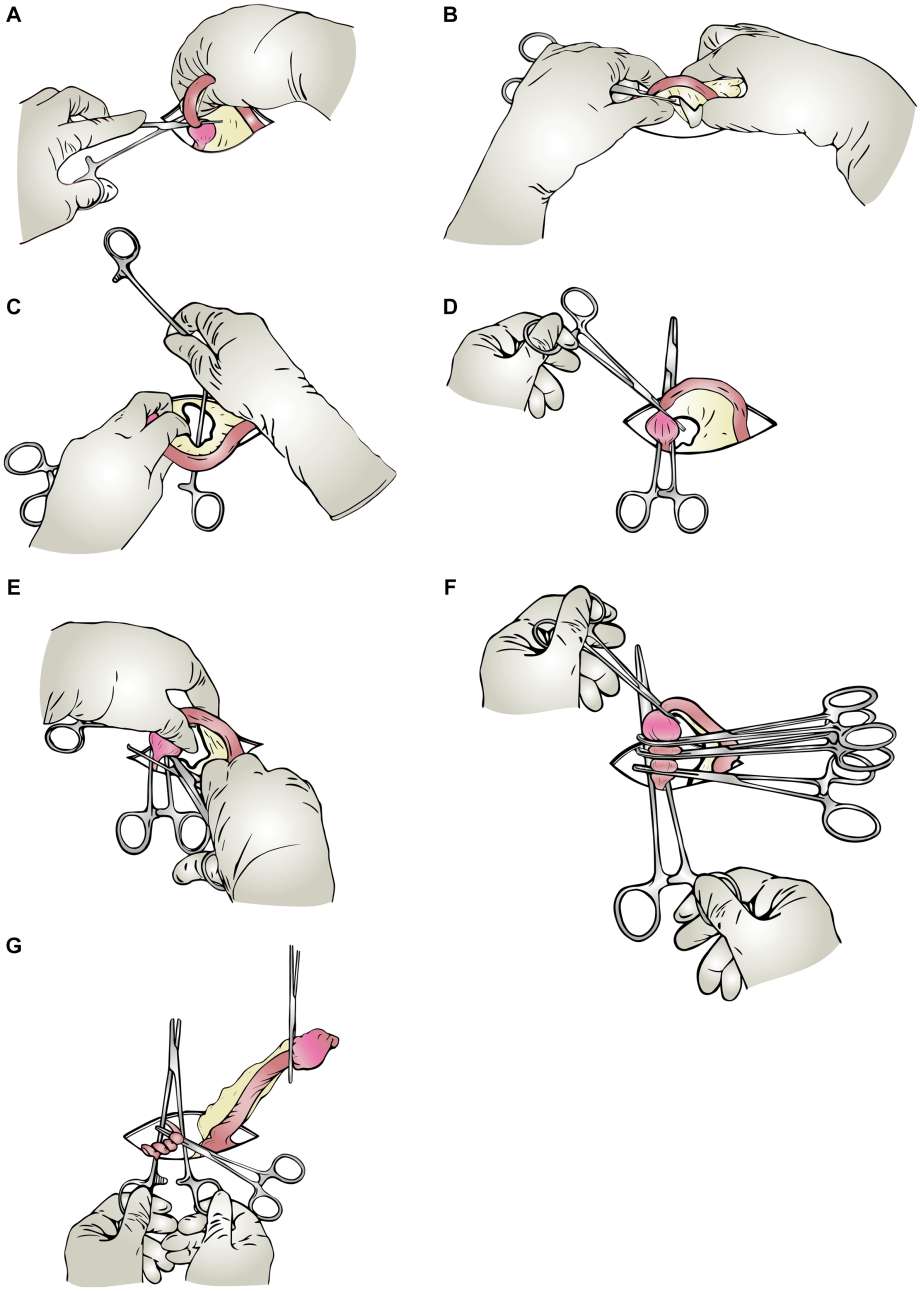
Schematic steps of the modified Instrument shank-assisted ovariohysterectomy. Most steps in this method are similar to the triple-clamp technique. Wherein the pedicle is retained outside the abdomen by a straight Ochsner hemostat placed on the shanks of a needle-holder crossly. **(A)** Proper ligament clamping: After identifying the uterine horn, the rat-toothed Crile forceps are placed on the proper ligament to manipulate the ovary and its pedicle. **(B)** Windowing: A window is created in the mesovarium with an index finger. **(C)** Shanking (needle holder’s shank insertion): The needle holder’s shank is passed through the created window in the previous step, and the ratchets are locked, so the ovarian pedicle locates between the shanks. **(D)** Shank-IN: The shanks are oriented crossly to the incision on the abdomen. **(E)** Applying the “Anchoring clamp” (The ovarian pedicle’s 1st clamp): Holding the proper ligament forceps with one hand, push the anchoring clamp (first clamp), which is on the needle holder’s shank, toward the viscera, and lock it on a suitable level of the ovarian pedicle. **(F)** Triple hemostatic: The “ligating clamps” were secured between the Anchoring clamp and the ovary out of the abdominal cavity to crush the tissue for ligature placement. **(G)** Ligation and shank-OUT: Apply two simple ligatures and one transfixing ligature close to the ovary in the crushed groove created in the previous step.

After accessing the visceral organs, first the left uterine horn and ovary were seized. A hemostat forceps was placed on the proper ligament to manipulate the ovary and its pedicle (Figure 1A). Then a window was created in the broad ligament (Figure 1B), and one of the handles of a Mayo-Hegar needle holder was passed through this window (Figure 1C), then secured (Figure 1D). After securing the ratchets, the needle holder was positioned over the surgical incision on the abdomen (Figure 1D). While pulling the first hemostat (on the proper ligament), the second hemostat was put on the ovarian pedicle, as far away from the ovary as possible (between the ovary and the needle holder’s locked handles) (Figure 1E). While doing this, with the second hemostat, the needle holder shanks were pushed along the suspensory ligament toward the viscera to attain the appropriate distance. After securing the second hemostat, it was placed crosswise on the needle holder’s shanks so that it would not be dragged into the abdominal cavity and would stay visible to the surgeon outside the abdomen at all times (Figure 1F). The third and fourth hemostats were then inserted between the ovary and the second forceps (Figure 1F). These forceps have crushed the tissues in preparation for the installation of the ligature. After crushing the pedicle, the forceps were removed, and circumferential and transfixation ligatures were applied to the pedicle in the formed groove. The ovarian pedicle was sharply transected using a scalpel immediately after the transfixation ligature, while it was protected by a hemostat. Throughout the application of these steps, the second forceps remained firmly on the ovarian pedicle, preventing it from being dragged within. The needle holder was put down after being released (Figure 1G). After gently grasping the corner of the ovarian pedicle with tissue forceps, the second forceps was released. The ovarian pedicle was inspected for hemorrhage and then released. The procedures were repeated for the contralateral ovary. The remainder of the procedure up to the final skin gap suture was routinely performed (same as the triple hemostatic method).

#### 2.3.5. Surgical time intervals

All surgeries were video captured, and the time intervals between each step were retrieved. The initiation of the incision and the final abdominal closure suture were considered the beginning and finish of the surgical procedure, respectively. These are the defined time intervals:

*Total surgery time* (TST): From the initial skin incision to the last skin suture of an abdominal incision.

*Pedicle intervention time* (PIT): From placing a hemostat on the proper ligament of the left ovary through cutting the right pedicle following the installation of its ligature; incorporating SRT, ShT, ClpT, and LigT for the left and right pedicles.

*Suspensory release time* (SRT): Digital strumming of the suspensory ligament.

*Shanking time* (ShT): From the end of windowing (the process of creating a window in the broad ligament) until the start of anchoring clamp placement. An *anchoring clamp* is a hemostatic clamp placed on the ovarian pedicle as far away from the ovary as possible to prevent dragging into the abdominal cavity.

*Clamping time* (ClpT): From the placement of the anchoring clamp to the completion of the last ligating clamp, near the ovary. The *ligating clamp* is a hemostatic forceps that is used to crush the ovarian pedicle and create a groove for the installation of the ligature. After the anchoring clamp, these forceps are secured to the pedicle on the ovary side.

*Ligating time* (LigT): From the beginning of the first simple ligature until the finish of the trans-fixing ligature of both pedicles.

*Other surgical procedures time* (OSPT): All surgical procedures, except PIT and CT.

*Closure time* (CT): Closure time starts from the linea alba to the last skin suture tying.

### 2.4. Pain assessments

There are many different kinds of subjective scoring systems, and some of them have been used in veterinary medicine (59). In this study, postoperative pain was measured using the Glasgow composite pain scale short form (GCMPS-SF) (60), the University of Melbourne pain scale (UMPS) (61), and visual analogue scales (VAS) (62). A trained, male examiner (AB) who was single-blinded measured the post-surgical pain (30, 31). He became acquainted with the dogs the day before surgery. Moreover, the similar abdominal closure and the bandage have prevented the procedure from being identified. Multidimensional pain assessments were carried out 1 h before surgery (0) and 6-, 24-, 48-, and 72-h following skin suturing.

### 2.5. CRP

Five milliliters of blood were drawn from the lateral saphenous vein after the same pain evaluation times. The samples were kept at the temperature of the operation room for 20–25 min before being transported to the laboratory. The serum separated by centrifuging at 3,000 rpm for 15 min was stored in a microtube at −20°C until the research ended. CRP levels (mg L^−1^) were determined using the CRP latex agglutination technique using the CRP-LIA kit, Bionik in the laboratory of the faculty (4, 33).

### 2.6. Postoperative medications

Antimicrobial therapy (63) was started intravenously (IV) during premedication and maintained intramuscularly (IM) every 8 h for 3 days following surgery at 22 mg kg^−1^ of cefazolin (Exir, Tehran, Iran) in both groups. Ketoprofen (Ketomax; Rooyandarou, Tehran, Iran) at 2 mg kg^−1^ IM was given to animals having a GCMPS-SF score of 6 or higher out of 24 (45); throughout the examination, the UMPS and VAS ratings were also examined as supplementary criteria for determining a ketoprofen prescription.

### 2.7. Statistical analysis

The SPSS program (IBM SPSS Statistics for Windows, version 26, IBM Corp., Armonk, NY, United States) was used to look at the data. Along with normality confirmation using the Shapiro–Wilk test (except LigT), the study recruited the suspicious non-normal parametric data (PID, SRT, ShT, ClpT, LigT, and OSPT) after normalization based on the concordance of skewness and kurtosis coupled with stem-and-leaf plots. The duration of surgical steps was analyzed using a *T*-test. The Pearson correlation coefficient was used to determine the relationship between PIT and TST.

The Friedman test analyzes the progression of pain changes. Using Related-Samples Friedman’s Two-Way ANOVA by Ranks, we compared pain levels between evaluating time intervals throughout each surgical process. Using Kruskal-Wallis H tests on mean, a comparison of pain levels at assessing time intervals between two surgical techniques has been conducted. On the median, Independent-Samples Kruskal-Wallis H followed by Independent-Samples Fisher Exact Sig. (2-sided test, for samples less than 10) has been implemented on the information obtained from three behavioral pain assessment methods.

Variations in CRP were evaluated using repeated measures ANOVA, independent samples t-test, and general linear model-univariate tests.

The correlation between CRP and age, weight, HR, RR, and RT was shown using Pearson’s correlation coefficient. The partial eta squared was used to figure out how CRP and the body condition score (BCS) are related. Spearman’s rho correlation coefficient was used to determine the relation between surgical time intervals and pain (based on GCMPS-SF). The statistical significance level was set at *p* < 0.05.

#### 2.7.1. Data availability statement

All relevant data is contained within the article: The original contributions presented in the study are included in the article, further inquiries can be directed to the corresponding author.

## 3. Results

### 3.1. Participants’ signalment and vital sign

There were no significant differences in the distribution of dogs by weight (*p* = 0.726) or age (*p* = 0.598) between the two groups. The mean and SD of age, weight, vital parameters (HR, RR, and RT), and BCS are shown in Table 2.

**Table 2:** Mean ± SD of age, weight, BCS, and preoperative vital parameters before anesthesia for the conventional (*n* = 9) and Instrument Shank-assisted ovariohysterectomy (*n* = 9).

Parameters	Conventional	Shank-assisted	*p-*value
Age	3.0 ± 1.9	2.6 ± 1.0	0.821
Weight	22.6 ± 4.3	21.8 ± 4.5	0.798
RT	38.7 ± 0.6	38.7 ± 0.3	0.877
HR	103.2 ± 25.0	106.0 ± 34.1	0.948
RR	29.3 ± 9.4	25.6 ± 5.0	0.622
BCS	4.7 ± 0.9	4.6 ± 0.9	0.422

BCS, body condition scoring; RT, rectal temperature; HR, heart rate; RR, respiratory rate. The significance level was set at 0.05.

### 3.2. Surgical time analysis

Instrument shank-assisted OHE displayed shorter TSTs than the conventional method (34.70 ± 6.7 and 47.40 ± 9.9 min respectively, *p* = 0.005). Table 3 provides a comparison of the methods’ time intervals. According to the new method, SRT is identical to ShT. ShT required 74% less time than SRT (*p* = 0.009), and moreover, LigT improved by 37% (*p* = 0.005) with the novel approach. Additionally, the OSPT and CT got 26 and 29% shorter, respectively.

**Table 3:** Mean ± SD of different surgical steps time intervals recorded in the conventional (*n* = 9) and Instrument Shank-assisted ovariohysterectomy (*n* = 9) and correlation of surgical time intervals with total surgery time in deep-chested dogs.

Data	Units	Conventional	Shank-assisted	*p-*value
Time intervals				
TST	min	47.40 ± 9.9	34.70 ± 6.7	0.005
PIT	min	20.96 ± 5.78	14.52 ± 3.73	0.014
SRT	s	78.97 ± 69.10		}0.035
ShT	s		20.39 ± 8.18	
ClpT	s	50.66 ± 45.04	63.55 ± 37.15	0.662
LigT	min	12.82 ± 3.37	8.02 ± 3.11	0.005
OSPT	min	31.00 ± 7.0	23.00 ± 4.8	0.013
CT	min	16.40 ± 4.5	11.60 ± 2.5	0.013

Correlation^*^				
	*ρ*	*p-*value	*ρ*	*p-*value
TST vs. PIT	0.952^****^	0.000	0.862^***^	0.003
SRT	0.494	0.176		
ShT			0.097	0.804
ClpT	0.683^**^	0.043	0.754^***^	0.019
LigT	0.836^***^	0.010	0.618	0.076
TST vs. CT	0.768^***^	0.016	0.818^***^	0.007

TST, total surgery time; PIT, pedicle intervention time; SRT, suspensory release time; ShT, shanking time; ClpT, clamping time; LigT, ligating time; OSPT, other surgical procedures time; CT, closure time. ^*^Pearson correlation stratification (very strong, strong, moderate, weak, and negligible): ^****^very strong, ^***^strong, ^**^moderate. The significance level was set at 0.05.

**Figure 2:**
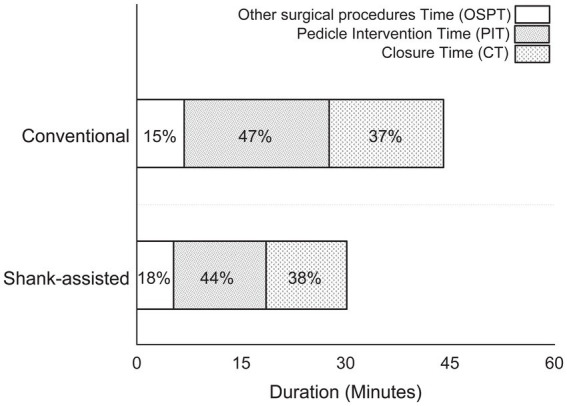
Comparison of the time spent performing different steps in the conventional method (*n* = 9) and new modified Instrument shank-assisted method of ovariohysterectomy (*n* = 9) in deep chested-dogs. OSPT, Other surgical procedures time, which consist of all surgical steps, except PIT and CT; PIT, Pedicle intervention time, which is from placing a hemostat on the proper ligament of the left ovary to cutting the right pedicle after placement of its ligatures; CT, Closure time, from the linea alba to the last skin suture tying.

Figure 2 shows how time intervals have changed, and Table 3 gives a statistical analysis of the changes. In each compartment of this figure, the difference favors the Instrument shank-assisted method.

According to the comparable correlation pattern (64) between TST and PIT based on ρ = 0.952, *p* = 0.0001 and ρ = 0.862, *p* = 0.003 for conventional and instrument shank-assisted OHE, respectively, PIT plays a crucial role in TST during ovariohysterectomy. The correlation analysis of TST and ShT revealed that, from a temporal perspective, ShT alone did not significantly reduce TST (*ρ* = 0.097, *p* = 0.804).

During the conventional OHE, a single intraoperative hemorrhage in the ovarian pedicle was managed. The dog was excluded from the study. None of the dogs in either group had problems after surgery.

### 3.3. Pain

**Figure 3:**
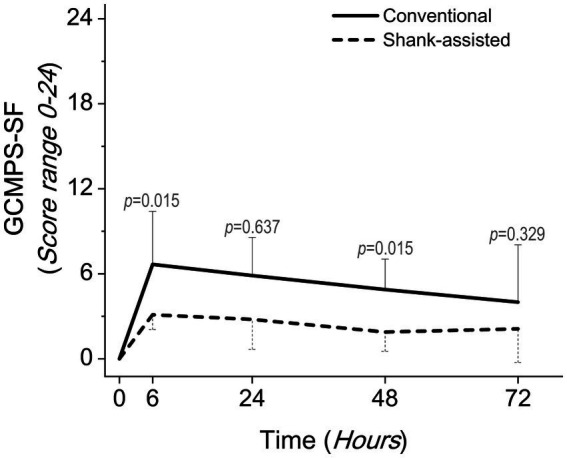
Comparing postoperative pain scores using the Glasgow composite pain scale short-form (GCMPS-SF) in the conventional method (*n* = 9) and new modified Instrument shank-assisted method (*n* = 9) of ovariohysterectomy in deep chested-dogs. The *p-*value was calculated using an Independent-Samples Kruskal-Wallis H on median followed by Independent-Samples Fisher Exact Sig. (2-sided test) on Median shows the statistical difference in sampling times between two groups.

**Figure 4:**
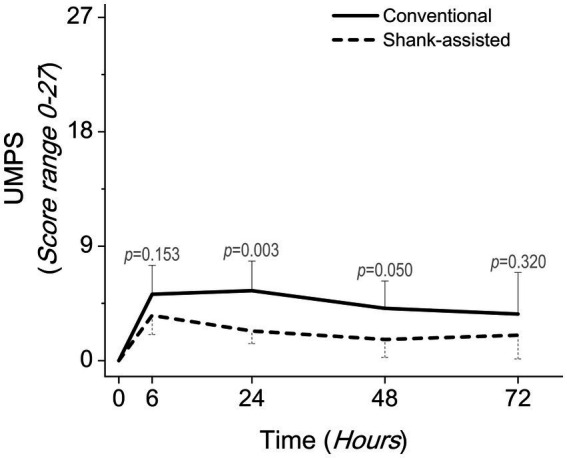
Comparing postoperative pain scores using the university of Melbourne pain scale (UMPS) in the conventional method (*n* = 9) and new modified Instrument shank-assisted method (*n* = 9) of ovariohysterectomy in deep chested-dogs. The *p-*value was calculated using an Independent-Samples Kruskal-Wallis H on median followed by Independent-Samples Fisher Exact Sig. (2-sided test) on Median shows the statistical difference in sampling times between two groups.

**Figure 5:**
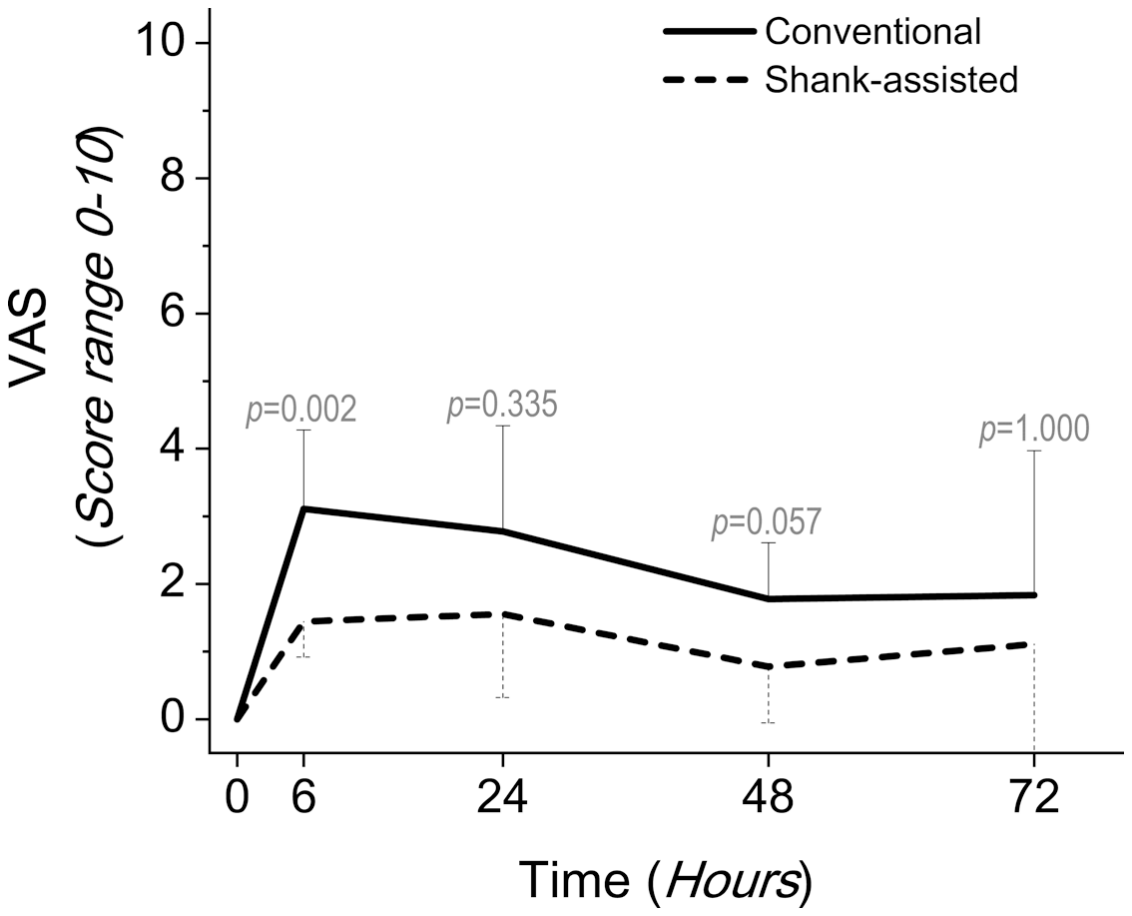
Comparing postoperative pain scores using the visual analogue scales (VAS) in the conventional method (*n* = 9) and new modified Instrument shank-assisted method (*n* = 9) of ovariohysterectomy in deep chested-dogs. The *p-*value was calculated using an Independent-Samples Kruskal-Wallis H on median followed by Independent-Samples Fisher Exact Sig. (2-sided test) on Median shows the statistical difference in sampling times between two groups.

Using the Friedman test, pain score changes (Δ Pain in GCMPS-SF, UMPS, and VAS; Table 3) were statistically significant in both groups. The majority of the time, the data showed that the new method was associated with much less pain (Table 4 and Figures 3–5).

**Table 4:** Median (Min–Max) of pain scores and Δ Pain^0–72^ based on Glasgow composite pain scale short-form (GCMPS-SF), university of Melbourne pain scale (UMPS), and visual analogue scales (VAS) scales in the conventional (*n* = 9) and Instrument Shank-assisted ovariohysterectomy (*n* = 9) in deep-chested dogs.

			T0	T6	T24	T48	T72	*p-*value^†^ (Δ Pain^0–72^)
GCMPS-SF								
		Conventional	0 (0–0) (9)	**6 (2–14) (9)**	5 (3–10) (8)	**5 (2–8) (9)**	3 (1–12) (6)	0.002
		Shank-assisted	0 (0–0) (9)	**3 (2–5) (9)**	2 (0–6) (9)	**2 (0–4) (9)**	1 (0–7) (9)	0.000
*p-*value	Mean	K-W^*^	1.000	0.012	0.037	0.006	0.167	
	Median	K-W^**^		0.004	0.486	0.004	0.264	
		Fisher^#^		**0.015**	0.637	**0.015**	0.329	
UMPS								
		Conventional	0 (0–0) (9)	5 (2–8) (9)	**5 (3–10) (8)**	**5 (0–7) (9)**	3 (1–10) (6)	0.003
		Shank-assisted	0 (0–0) (9)	4 (1–6) (9)	**2 (1–4) (9)**	**1 (0–4) (9)**	2 (0–5) (9)	0.000
*p-*value	Mean	K-W^*^	1.000	0.099	0.002	0.018	1.000	
	Median	K-W^**^		0.058	0.002	0.016	0.264	
		Fisher		0.153	**0.003**	**0.050**	0.329	
VAS								
		Conventional	0 (0–0) (9)	**3 (1–5) (9)**	3 (1–6) (9)	2 (0–3) (9)	1 (0–6) (6)	0.002
		Shank-assisted	0 (0–0) (9)	**1 (1–2) (9)**	1 (0–4) (9)	1 (0–2) (9)	0 (0–5) (9)	0.001
*p-*value	Mean	K-W^*^	1.000	0.003	0.074	0.024	1.000	
	Median	K-W^**^		0.001	0.147	0.018	1.000	
		Fisher		**0.002**	0.335	0.057	1.000	

T0, before surgery; T6, 6 h after surgery; T24, 24 h after surgery; T48, 48 h after surgery; T72, 72 h after surgery; eGCMPS-SF, the short form of the Glasgow composite measure pain scale; UMPS, university of Melbourne pain scale; VAS, visual analogue scale. The bolded items show a statistically significant difference in a pain score between the two surgical procedures in each evaluating time intervals using the Kruskal-Wallis H analysis; Lowercase letters indicate statistical comparison of pain recorded at different assessment times in the same group using Related-Samples Friedman’s Two-Way ANOVA by Ranks. ^†^Friedman Test. ^*^Independent-Samples Kruskal-Wallis H on mean. ^**^Independent-Samples Kruskal-Wallis H on median. ^#^Independent-Samples Fisher Exact Sig. (2-sided test) on Median (for samples less than 10). The significance level was set at 0.05.

#### 3.3.1. Rescue analgesia

After the surgery, 36 pain assessments have been done in each group, ranging from T6 to T72. These pain assessments have been carried out at 6-, 24-, 48-, and 72-h following surgery on nine animals in each group. The animals were injected with rescue analgesics 13 times (T6: 6, T24: 3, T48: 3, and T72: 1 dog) in the conventional group and twice (T24: 1 and T72: 1, both for one dog) in the novel group. Using the conventional methods, 8 dogs were injected with rescue analgesic, whereas just 1 dog received it using the alternative method.

### 3.4. CRP

**Figure 6:**
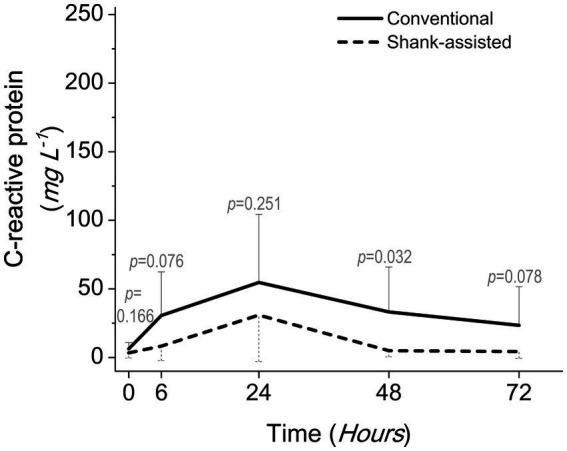
Comparing postoperative serum C-reactive protein levels following the conventional method (*n* = 9) and new modified Instrument shank-assisted method (*n* = 9) of ovariohysterectomy in deep chested-dogs. The *p-*value was calculated using an independent samples *T*-test shows the statistical difference in sampling times between two groups.

After 24 h, the highest serum CRP levels were observed in both groups. Figure 6 demonstrates that the conventional group’s rate of rise accelerated more rapidly during the initial 6 h following surgery. The slope of the graph is determined to be *y* = 24.328x − 18.072 for traditional OHE and *y* = 4.9556x − 1.5667 for instrument shank-assisted OHE within the first 6 h. The traditional group observed a 4.9-fold increase in CRP acceleration on this basis. After 48 h, the Instrument shank-assisted OHE showed a significant decrease to baseline levels (*p* = 0.032; see Tables 5, 6). In contrast, although CRP levels decreased in the conventional group, they were not significantly different from their peak levels.

**Table 5:** Mean ± SD of the serum CRP concentration (mg L^−1^) following the conventional (*n* = 9) and Instrument Shank-assisted ovariohysterectomy (*n* = 9) in deep-chested dogs.

Sampling time	Groups		*p-*value
	Conventional	Instrument shank-assisted	
T 0	6.3 ± 4.6^b^	3.4 ± 3.8^b^	0.166
T 6	30.6 ± 31.9^ab^	8.3 ± 10.7^b^	0.076
T 24	54.7 ± 49.6^a^	30.9 ± 33.7^a^	0.251
T 48	33.2 ± 32.7^ab^	5.0 ± 4.4^b^	**0.032**
T 72	23.4 ± 28.2^ab^	4.4 ± 5.0^b^	0.078

Small letters indicate statistical comparisons in a group between different sampling times. The *p*-value shows the comparison of two groups at each sampling time. T 0, before surgery; T 6, 6 h after surgery; T 24, 24 h after surgery; T 48, 48 h after surgery; T 72, 72 h after surgery. The significance level was set at 0.05. *p*, *p*-value, the bolded text shows a significant correlation.

### 3.5. Correlations

The relationship between pain (as measured by GCMPS-SF, UMPS, and VAS), surgical time parameters, CRP, vital signs, age, weight, and BCS has been studied.

#### 3.5.1. Surgical time intervals with pain

Spearman’s rho correlation coefficient has studied the relationship between the overall surgical duration and the duration of the distinct phases. The analysis is described in full in Table 6, which is stated separately below.

**Table 6:** The correlation of surgical time intervals with post-surgical pain, which was evaluated using GCMPS-SF, UMPS, and VAS behavioral pain scales, after the conventional (*n* = 9) and Instrument Shank-assisted ovariohysterectomy (*n* = 9) in deep-chested mixed-breed dogs.

Pain scales	Surgical time intervals	Methods															
		Conventional								Instrument shank-assisted							
		Pain 6		Pain 24		Pain 48		Pain 72		Pain 6		Pain 24		Pain 48		Pain 72	
		ρ	*p*	ρ	*p*	ρ	*p*	ρ	*p*	ρ	*p*	ρ	*p*	ρ	*p*	ρ	*p*
GCMPS-SF	TST	0.254	0.509	0.268	0.520	0.246	0.524	0.754	0.084	0.039	0.920	−0.343	0.366	−0.489	0.181	−0.309	0.418
	PIT	0.085	0.828	0.024	0.954	0.246	0.524	0.638	0.173	−0.208	0.591	−0.270	0.482	−0.282	0.462	−0.162	0.676
	SRT/ShT	0.356	0.347	0.098	0.818	0.042	0.914	0.116	0.827	0.321	0.400	0.405	0.279	0.068	0.861	0.359	0.343
	ClpT	−0.254	0.509	−0.073	0.863	0.314	0.411	0.493	0.321	−0.009	0.982	−0.555	0.121	−0.622	0.073	−0.373	0.322
	LigT	−0.034	0.931	0.073	0.863	0.610	0.081	0.899^*^	**0.015**	−0.303	0.428	−0.523	0.148	−0.316	0.407	−0.368	0.330
	CT	0.237	0.539	0.464	0.247	0.398	0.288	0.725	0.103	0.390	0.300	−0.093	0.812	−0.385	0.307	−0.111	0.776
	OSPT	0.254	0.509	0.293	0.482	0.271	0.480	0.754	0.084	−0.091	0.815	−0.449	0.225	−0.541	0.133	−0.352	0.353
UMPS	TST	0.118	0.762	0.393	0.336	0.220	0.569	0.348	0.499	−0.521	0.150	−0.705^*^	**0.034**	−0.670^*^	**0.048**	−0.336	0.376
	PIT	−0.068	0.863	0.417	0.304	0.458	0.215	0.522	0.288	−0.434	0.243	−0.685^*^	**0.042**	−0.385	0.307	−0.180	0.642
	SRT/ShT	0.135	0.729	0.528	0.179	0.441	0.235	0.319	0.538	−0.230	0.552	0.035	0.929	0.034	0.930	0.395	0.293
	ClpT	−0.051	0.897	0.246	0.558	0.186	0.631	0.319	0.538	−0.427	0.251	−0.557	0.119	−0.588	0.096	−0.220	0.570
	LigT	0.152	0.696	0.442	0.273	0.576	0.104	0.696	0.125	−0.085	0.828	−0.416	0.266	−0.299	0.434	−0.335	0.378
	CT	0.397	0.291	0.466	0.244	0.203	0.600	0.377	0.461	−0.655	0.055	−0.503	0.168	−0.590	0.094	−0.129	0.741
	OSPT	−0.034	0.931	0.405	0.319	0.407	0.277	0.638	0.173	−0.402	0.284	−0.613	0.079	−0.644	0.061	−0.323	0.396
VAS	TST	0.254	0.510	0.368	0.330	−0.059	0.879	0.638	0.173	−0.522	0.150	−0.422	0.258	−0.622	0.074	−0.193	0.618
	PIT	−0.009	0.982	0.368	0.330	0.218	0.573	0.880^*^	**0.021**	−0.433	0.244	−0.329	0.388	−0.472	0.199	−0.110	0.778
	SRT/ShT	0.289	0.451	0.564	0.113	0.564	0.113	0.698	0.123	−0.173	0.656	0.310	0.416	−0.027	0.946	0.578	0.103
	ClpT	0.149	0.703	0.197	0.612	0.149	0.703	0.395	0.439	−0.696^*^	**0.037**	−0.688^*^	**0.041**	−0.751^*^	**0.020**	−0.484	0.187
	LigT	0.035	0.929	0.291	0.448	0.287	0.454	0.820^*^	**0.046**	−0.346	0.361	−0.566	0.112	−0.330	0.386	−0.523	0.149
	CT	0.455	0.219	0.197	0.612	0.010	0.980	0.577	0.231	−0.520	0.152	−0.402	0.284	−0.508	0.163	0.000	1.000
	OSPT	0.009	0.982	0.616	0.078	0.149	0.703	0.941^**^	**0.005**	−0.435	0.242	−0.394	0.294	−0.720^*^	**0.029**	−0.304	0.426

ρ, Spearman’s rho correlation coefficient; *p*, *p*-value, the bolded text shows a significant correlation; Pain 6, pain assessment 6 h after surgery; Pain 24, pain assessment 24 h after surgery; Pain 48, pain assessment 48 h after surgery; Pain 72, pain assessment 72 h after surgery; GCMPS-SF, the short form of the Glasgow composite measure pain scale; UMPS, university of Melbourne pain scale; VAS, visual analogue scale; TST, total surgery time; PIT, pedicle intervention time; SRT, suspensory release time; ShT, shanking time; ClpT, clamping time; LigT, ligating time; OSPT, other surgical procedures time; CT, closure time. ^*^Correlation is significant at the 0.01 level (2-tailed). ^*^ Correlation is significant at the 0.05 level (2-tailed).

Only pain in the conventional OHE exhibits a significant positive correlation with LigT at T72, according to GCMPS-SF.

Using the novel method, UMPS found significant negative relationships between pain and TST at T24 and T48. This study indicated that PIT has a vital function in lowering pain in T24.

The VAS has found a greater correlation between specific surgical stages and pain. GCMPS-SF, UMPS, and VAS were able to identify 1, 3, and 7 correlations, respectively, in this regard. In the new method, VAS identified an association between ClpT and less pain at T6 and T24, and between ClpT and OSPT at T48. This assessment method revealed that PIT, LigT, and most notably OSPT have a substantial effect on the incidence of pain on the third day following surgery in the conventional group (see Table 6).

#### 3.5.2. CRP with pain

Using Spearman’s rho correlation coefficient, CRP levels and pain were only shown to have significant moderate-to-strong negative relationships in five measurement points of total samples (two groups in total). These associations were detected at T24 (UMPS) and T72 (GCMPS-SF) in the conventional group and at T6 (VAS) and T48 (GCMPS-SF and VAS) in the instrument shank-assisted group, as shown in Table 7.

**Table 7:** The correlation of serum c-reactive protein levels with post-surgical pain, which was evaluated using GCMPS-SF, UMPS, and VAS behavioral pain scales, after the conventional (*n* = 9) and Instrument Shank-assisted ovariohysterectomy (*n* = 9) in deep-chested mixed-breed dogs.

Pain scales	CRP sampling times	Methods															
		Conventional								Instrument shank-assisted							
		Pain 6		Pain 24		Pain 48		Pain 72		Pain 6		Pain 24		Pain 48		Pain 72	
		ρ	*p*	ρ	*p*	ρ	*p*	ρ	*p*	ρ	*p*	ρ	*p*	ρ	*p*	ρ	*p*
GCMPS-SF	CRP 6	0.593	0.092	0.475	0.197	0.458	0.215	0.407	0.277	−0.182	0.639	−0.459	0.214	−0.130	0.739	−0.303	0.428
	CRP 24	−0.049	0.909	0.146	0.729	−0.195	0.643	−0.122	0.774	−0.498	0.173	−0.034	0.931	0.143	0.713	0.143	0.713
	CRP 48	−0.576	0.104	−0.280	0.466	−0.509	0.162	−0.492	0.179	−0.718^*^	**0.029**	−0.085	0.827	−0.051	0.896	0.043	0.913
	CRP 72	−0.551	0.257	−0.493	0.321	−0.638	0.173	−0.812^*^	**0.050**	−0.462	0.211	0.000	1.000	0.188	0.628	0.248	0.520
UMPS	CRP 6	0.245	0.526	0.304	0.427	0.321	0.400	0.152	0.696	−0.511	0.160	−0.034	0.931	−0.085	0.828	−0.111	0.777
	CRP 24	−0.626	0.097	−0.356	0.387	−0.638	0.089	−0.724^*^	**0.042**	−0.373	0.323	0.121	0.756	−0.243	0.529	−0.225	0.560
	CRP 48	−0.441	0.235	−0.153	0.695	−0.356	0.347	−0.356	0.347	−0.616	0.078	−0.068	0.861	−0.103	0.793	0.009	0.983
	CRP 72	−0.522	0.288	0.145	0.784	0.029	0.957	−0.058	0.913	−0.326	0.391	−0.034	0.930	0.198	0.610	0.240	0.533
VAS	CRP 6	0.140	0.720	0.009	0.982	0.114	0.771	−0.035	0.929	−0.779^*^	**0.013**	0.000	1.000	−0.087	0.825	−0.087	0.825
	CRP 24	−0.231	0.550	−0.043	0.913	−0.316	0.407	−0.162	0.676	−0.256	0.507	0.402	0.284	0.383	0.308	0.347	0.360
	CRP 48	−0.337	0.376	0.050	0.899	−0.069	0.859	−0.139	0.722	−0.713^*^	**0.031**	−0.160	0.680	−0.267	0.487	−0.205	0.597
	CRP 72	−0.577	0.231	−0.152	0.774	−0.213	0.686	−0.334	0.518	−0.220	0.569	0.303	0.428	0.440	0.235	0.468	0.204

ρ, Spearman’s rho correlation coefficient; *p*, *p*-value, the bolded text shows a significant correlation; CRP, c-reactive protein; CRP 6, CRP sampling 6 h after surgery; CRP 24, CRP sampling 24 h after surgery; CRP 48, CRP sampling 48 h after surgery; CRP 72, CRP sampling 72 h after surgery; Pain 6, pain assessment 6 h after surgery; Pain 24, pain assessment 24 h after surgery; Pain 48, pain assessment 48 h after surgery; Pain 72, pain assessment 72 h after surgery; GCMPS-SF, the short form of the Glasgow composite measure pain scale; UMPS, university of Melbourne pain scale; VAS, visual analogue scale. ^*^Correlation is significant at the 0.05 level (2-tailed).

#### 3.5.3. CRP with age, weight, vital signs, and BCS

Pearson’s correlation coefficient indicated that there was no association between CRP changes and age, weight, HR, RR, and RT in both groups (*p* > 0.05). Partial eta squared (η P 2) was unable to identify a significant association between CRP and BCS using any of the two techniques.

## 4. Discussion

According to the present results, when implementing OHE with instrumental shank assisted technique, the surgery can be performed by one person with lesser surgical trauma. With the current solutions, digital strumming or sharp tearing of the suspensory ligament in deep-chested dogs has not only been a time-consuming process, but it has also failed to shorten the length of surgery and pain afterward (11). According to current research, a decrease of over 12 min in an alumnus surgeon’s overall surgery length is a positive improvement (57). The reduction in surgical trauma has resulted in a slower increase in CRP levels and a shorter peak. These gains were made using the same equipment and facilities at no additional expense due to a modest modification in the surgical approach.

### 4.1. Surgical perspectives

OHE, like many other surgical procedures, seems to get more challenging as the size and weight of the animal increase. As body mass increases, the chest sinks deeper, and it becomes more difficult to reach and release the suspensory ligament (11). In the present study, the same higher body weight range enhanced the ovarian exposure challenge, allowing the method to be generalized to various sizes of dogs and cats; however, it may be accompanied by fewer problems in smaller animals.

Insufficient exposure during OHE leads to incorrect technique execution and raises the risk of ovarian remnant syndrome (2). The instrument shank-assisted OHE keeps the ovary outside of the abdomen without an assistant while maintaining the suspensory ligament. In obese and deep-chested dogs, as well as for unskilled surgeons who require a longer incision, the surgical assistant is essential (1, 2). So, a small incision without tearing the ligament is another achievement of the modified method, which led to limited surgical complications including incisional swelling, seroma, infection, delayed healing, ventral body wall dehiscence, self-inflicted trauma, pain (65), and hemorrhaging (66).

During a suspensory ligament release (66), an inexperienced surgeon is more likely to break the blood vessel and induce hemorrhage, which prompts the hurried application of surgical sponges. Stress has a negative impact on the non-technical skills of surgeons (67), leading Rodriguez et al. to conclude that intraoperative hemorrhage from an ovarian pedicle probably increased the retention of surgical sponges in veterinary patients (68). Therefore, removing ligament release from the surgical steps would likely reduce the frequency of hemorrhage and sponge retention; however, more research is required before a conclusion can be reached.

Hilgard’s learning theory suggests that experience is a crucial component of the learning process (69). For unbiased comparisons, the study surgeries must be performed by a surgeon with no or equivalent prior experience in both methods. A minimum of surgeon experience in our study to the level of the veterinary training program according to conventional method seems to impose an inevitable minimal bias. However, the presence of an inexperienced surgeon could be a limitation of the present study. Given the possibility that a surgeon’s lack of expertise might exacerbate surgical stress to the point where the impact of the technique is nullified, a week of training for each method prior to the research allowed the current study to reveal the smallest difference between the procedures. TST has been reported to take between 55 and 130 min for inexperienced surgeons (70, 71). Freeman et al. established an optimal duration of 45 min for inexperienced surgeons after six surgeries (70). In this study, an average of almost 40 min TST demonstrated that the surgeon’s skill is enhanced by training before to the start of main operations, and demonstrating our surgeons’ experience (71) made the findings more realistic (11, 13, 70). Alternatively, previous research on pilots has yielded five levels of skill acquisition, including novice, advanced beginner, competent, proficient, and expert (25); If each 10-min improvement for OHE corresponded to one level of improvement for the surgeon’s expert, then our surgeon’s 12.7 min reduction in surgical duration using the instrument shank-assisted technique would theoretically qualify her as “competent.”

Surgical experience makes sick animals healthier (72). Sir Francis Galton thought that talent was completely innate (73), but experience is a learning growth (25). Yet, from a different perspective, the surgeon’s experience may provide no more than a 25% health improvement (72). On the other hand, OHE is still known as a model of acute pain in research studies. So, when animal pain after a technique is still a problem even after experienced surgeons have used it, it is important to look at the technique itself instead of just how it is taught. Consequently, both correct training and the training of correct techniques are emphasized in the surgical training curriculum, and the new method may contribute to the promotion of the latter, as indicated by the high effect sizes reported in the present study.

The type of operation is a stressor for the surgeon (74). Mental (75) and muscle (76) fatigue can delay an operation. Aside from the fact that the stress was not directly evaluated in our study, time as a major component in calculating surgical stress (74, 77) has been meticulously recorded and analyzed. Controversial is the scenario in which the total surgery time is reduced beyond the time spent on the ovarian pedicle. The technical difference between the two methods was SRT and ShT only had a time difference of 58.58 s in favor of the modified method, but TST was improved by 12.7 min. Non-correlation of these variables with TST indicates they did not directly contribute to the difference in TST, while ovarian exposure caused roughly 63% quicker application of ligatures (4.8 min improvement). The remainder of the improved duration was divided into two parts: (1) 3.1 min from the major procedures in OSPT, which include the separation of the broad ligament on both sides, the second ovary access, uterine arteries ligature placement, and uterine body close and cut; and (2) 4.8 min from CT. The procedures conducted in OSPT and CT were comparable among techniques, although the Instrument shank-assisted OHE required significantly less time. When we were nearing the end of the surgery, or, in other words, when the surgeon had reached extreme fatigue, 4.8 min more time was required to close the abdominal wall using the conventional technique. Therefore, significant time reduction in the mentioned two parts could be related to less fatigue and stress, which was paved through the shorter LigT in the new method. Peeters and Kirpensteijn’s unsuccessful attempt to reduce surgical time by utilizing ovariectomy (OVE) instead of OHE (58) is another example of the surgeon’s mental and physical strain at this time, as they did not eliminate digital strumming. Due to the strong correlation between TST and LigT in the present study, it is evident that the modified instrument shank-assisted technique can reduce the impact of the time required to install the ligatures, which was the primary factor in the significantly longer surgical time when the previous technique was used. Additionally, the small standard deviation (78) in these two portions may indicate the surgeon’s optimal state of stability and fewer technical obstacles during instrument shank-assisted OHE, which may require further investigation.

According to research findings, the surgeon’s stress level may be enhanced due to a lack of familiarity with the members of the surgical team (67). While the present study did not measure the stress level of the surgeon, efforts were made to mitigate concerns regarding the presence of new team members. This was achieved through measures such as facilitating familiarity and collaboration among team members during the pre-study training course as well as maintaining a consistent surgical team.

Surgical supervisor changes complicate student-led surgical procedures (57). This study’s experienced academic surgeon continuously supervised the surgical procedures, eliminating the possibility of this error, as observed in Harris’s study due to a change in supervisor.

The time interval proportion found in this study could be used in general, even though an experienced surgeon could cut the total time needed for surgery. Hence, based on Shivley’s yearly savings of 73.3 h (11), a 26.7% decrease in TST in instrument shank-assisted OHE could save 195.7 h (>2.5 times).

In concluding, as ovarian manipulation and pedicle ligatures were identified as the most essential procedures of OHE in a previous study (79), these findings have been meticulously confirmed in the present study. In the instrument shank-assisted OHE, a more accessible ovarian pedicle facilitated all subsequent steps of the surgery.

Due to the surgical discussion’s focus on inexperienced surgeons, it is reasonable to assume that the significance of this technique will change as the surgeon’s experience grows, whereas the pain-related advantages of this surgical technique will be discussed in a separate chapter, considering all surgeons to be subject to the implementation of this technique.

### 4.2. Pain perspective

The current study confirmed that, compared to instrument shank-assisted OHE, digital strumming of the ovarian pedicle during conventional OHE makes ligature placement more challenging, possibly due to unwanted visceral interferences or manipulations, prolongs the duration of surgery, and increases pain. Digital strumming is an unpleasant surgical procedure followed by considerable surgical trauma. Thereby, it is anticipated that modifying traditionally invasive procedures would improve patients’ recoveries and well-being.

Subjective pain scales (44, 80) and, controversially, CRP have been used to measure pain since vital signs are not sensitive enough (81) and animals cannot communicate verbally (47). Several multidimensional structured behavior scales have been adapted for use in veterinary medicine (59), and the multidimensional GCMPS-SF for acute pain has been authorized for use in dogs (45, 60) with more sensitive and consistent results (82). The UMPS (which comprises six categories of physiological data) and VAS (with more flexibility) were used to compensate for the insufficiency of the GCMPS-SF and reduce the secrecy of the pain.

The results of pain assessments vary based on the experience and knowledge of the veterinarians, which are affected by age, gender, and time since graduation (31, 83). It was anticipated that using a trained, blinded assessor (30), a wound dressing, and three distinct pain scales would reduce the influence of qualitative variable bias in the current study. On the other hand, demographic data and dog acclimation before surgery suggest that individual pain tolerance, species, age, body condition, and environmental factors that can change or mask pain intensity are not confounding variables.

Since severe pain after surgery is often underestimated (84), it needs to be measured in a new surgical procedure (85). The perception of postoperative pain is dependent not only on surgical duration and technique (86), but also on analgesic type (87), dose, multimodality (88–90), the use of preventative analgesia (91), route of administration, and the pharmacokinetics of medications (92). Pain can result in delayed wound healing and surgical site infection (93), and bandages may not be adequate for preventing suture line contamination (94). So, surgeons prefer to modify surgical procedures to reduce postoperative pain (47). This was one of the most important goals of instrument shank-assisted OHE, which had an effect size d of >1.27, > 1.32, and > 1.77 and a power of 0.811, 0.836, and 0.968 at T6, T24, and T48, respectively, based on provided GCMPS-SF pain measurements.

Sampling time is essential for accurately determining pain on time. In this study, sampling times were adjusted according to four theoretical elements: (1) the clinical duration of action of the analgesic agent (meperidine, medetomidine, and ketorolac), which may provide enough pain relief; (2) the plasma half-life of the anesthetic selected for premedication, induction, and maintenance of anesthesia (acepromazine, midazolam, and ketamine), which may change the responses given for pain evaluation; (3) the minimum estimated duration for pain onset, peak, and subsidence; and (4) the pathophysiology of CRP turnover. By giving dogs nonsteroidal anti-inflammatory drugs (NSAIDs) before surgery to help with pain after surgery (95, 96), it was thought that ketorolac might be enough for the first few hours after surgery. However, the results have only shown that this is true for the new technique. It was anticipated that the pharmacologic effects of the long-half-life (t_½_) drugs acepromazine (97), ketorolac (98, 99), and ketamine (nor-ketamine) (100) with residual effects of 7.1, 4.5 (or 10 based on relevant reference), and 6.2 h, respectively, would be felt from premedication until endotracheal extubation. Because sedatives, analgesics, and injectable dissociative anesthetics can change responses like facial expression, salivation, mydriasis, and cardiorespiratory parameters, which were evaluated in the GCMPS-SF, UMPS, and VAS, early pain assessment (<6 h) was not considered in this study.

The requirement of rescue analgesia may be regarded as a reliable indicator of the surgical technique’s incompetence. According to a previous report in humans (101), after hip and knee arthroplasty, ketoprofen had the same analgesic effect as extradural morphine. Mathews et al. find that ketoprofen has a comparable impact to meloxicam and conclude that it could be a useful way for controlling postoperative pain (102). Similar to carprofen, meloxicam, and tolfenamic acid, ketoprofen produced excellent postoperative analgesia in cats, but with a lesser effect on tenderness (103). In the meantime, more recent studies indicate the administration of NSAIDs is superior to opioids due to faster recovery of normal functions and greater satisfaction with postoperative well-being (104). Nevertheless, these findings should be taken with care when applied to OHE in veterinary medicine. In the present study, animals that got rescue analgesia were not excluded, and 8 dogs in the conventional group who received rescue analgesia at 13 evaluation times were included for analysis. If getting rescue analgesics improved outcomes, the dogs in the first group were unable to demonstrate superior outcomes despite receiving frequent pain treatment. The number of animals administered rescue analgesics may be a reflection of the severity of surgical trauma and the invasiveness of the conventional technique. Receiving rescue analgesics in 1 dog out of 2 evaluation times can be attributed to greater well-being using the modified method and can be interpreted in two aspects: first, a standard protocol of analgesics is still recommended after surgery, and second, it may be useful in shelter or stray dogs that may not receive proper follow-up treatment, for example.

At 6 h postoperatively, eight out of nine dogs treated with the conventional method received rescue analgesia. This demonstrates at least two important points: (1) ketorolac provides inadequate postoperative analgesia for the conventional OHE performed by an inexperienced surgeon, although it has been used in humans to control moderate to severe post-operative pain, and it may be effective in dogs (105), as effective as flunixin, and more effective than butorphanol or a low dose of oxymorphone (106), by affecting opioid receptors centrally with comparable efficacy to morphine (99), and (2) the current modification has decreased postoperative pain to the point that ketorolac could control it, so that none of the dogs in the second group required T6 rescue analgesia.

Our “competent” surgeon has made the surgery quick and competitive in terms of time (see “Surgical perspective”). Since the new method causes less pain and this surgery is still done to create a model of acute pain for research, it is safe to assume that this surgical method is not limited to a certain group of surgeons, no matter how much experience they have, and that it is better to make it more general.

In addition to the psychological burden experienced by the surgeon, contemporary approaches have been developed to assess the degree of pain and surgical stress imposed on the patient. While certain methods, including pupillometry, surgical pleth index (SPI), skin conductance, cardiovascular, and cardiorespiratory indices, require further advancements in sensor technology and interpretation algorithms to investigate animal responses to anesthesia and surgery, their applicability has not yet been confirmed. In the field of veterinary medicine, additional quantitative techniques have been introduced for animal assessment. These include the parasympathetic tone activity index (PTA index), which analyzes heart rate variability, and the bispectral index (BIS), which analyzes electroencephalography, with the potential for animal interpretation. Despite ongoing debates regarding the universal implementation of their use in all treatment procedures and drug protocols (107), the accessibility of these remedies may not always be assured. It is worth noting that the application of PTA as a means of assessing pain in conscious animals within the field of veterinary medicine is challenging. This is primarily due to the presence of unwanted movements by the animal, which directly impact heart rate variability. Consequently, alternative models capable of detecting these fluctuations should be employed (20, 32, 107, 108). The use of more recent medications, and therefore the recording of the quantity of anesthetics used (55) and the vital parameters throughout the operation, seems to be a large issue that would need review in separate research, other than that this technology was not available for the current study.

A number of different physiological parameters, such as plasma vasopressin, urine noradrenaline, and creatinine concentrations, have been suggested to assess the degree of irritation and pain caused by a surgical method. It appears that documenting additional facts and aiding in the final assessment of the effectiveness of the presented technique may be accomplished by comparing the changes in these parameters during anesthesia between the two methods. The conventional OHE involves applying extremely stretching stress to digitally strumming the suspensory ligament, while the new method involves keeping this ligament under tension all the way through the ligature placement process. Further research is warranted to compare the two methods from this perspective, as acute noxious stimuli during stretching of the pedicles can increase systolic blood pressure, heart rate, plasma vasopressin concentration, and urinary noradrenaline/creatinine ratio (55).

One of the initial observable events following surgery is the elevation in temperature and inflammation of the surgical site, attributed to enhanced blood circulation to the area where surgery was performed. Infrared thermography (IRT) is a proficient technique for assessing thermal variations in problematic areas, as it measures the surface temperature of the skin through thermographic maps. This technique may be used to recognize the localized changes in blood supply and localized increases in temperature that occur in response to stress. The assessment of the efficacy of local anesthesia through the analysis of alterations in surface blood circulation linked to sympathetic activity is among the additional functionalities of IRT (109). However, it has been observed that this technique has not demonstrated sufficient effectiveness in dogs (110). The present study suggests that while the implementation of IRT for evaluating pain and inflammation in the surgical approach area was effective, practical limitations arose due to the bandage covering the surgical site. Conversely, the thermographic assessment of regions where the suspensory ligament has been torn, situated on the roof of the abdominal cavity, may not be deemed reliable in theory. This is due to the fact that the heating of the dermis surface is directly linked to the local dermal microcirculation, which is under the control of the ANS. Nevertheless, the non-invasive nature of this method of evaluation may be an appealing subject for further research.

Pupil shape has been a key indicator for neurological assessment for over a century (111), and automated pupillometers have become increasingly important due to the difficulty of detecting the “reactive pupil” characteristic (112). In addition, assessing pupil reactivity using a pupillometer offers an objective, rather than subjective, evaluation of the neurological examination. Automated pupillometers can distinguish between canine conscious and anesthetic pupillary light reflex (PLR) and continuously assess an animal before, during, and after anesthesia (113). It seems that PLR devices could be useful in research like the current one, provided that the assessments are standardized.

Evaluation of a novel surgical procedure extends beyond surgical parameters and postoperative discomfort. Surgical invasiveness may be assessed separately. Based on past research, the invasiveness of the surgical procedure may be related to postoperative pain. Consequently, the question of surgical trauma severity is a separate topic that will be addressed further in “CRP perspective.”

### 4.3. CRP perspective

In this study, the traditional OHE method was changed in a way that reduced the amount of trauma. With the new technique, the amount of surgery-related trauma had a smoother pattern and went back to normal almost 30% faster. These results predicted a shorter period of recovery time subsequent to instrument shank-assisted OHE.

The postoperative acute-phase response develops faster in dogs compared to humans. Tissue damage and pain after invasive surgery are associated with a rise in blood acute-phase proteins, primarily CRP, which may be a valuable diagnostic biological marker of early postoperative complications (114, 115). The CRP, a sensitive biomarker of infection (116), inflammation, and tissue damage (46), is more sensitive than serum cortisol (81) in detecting surgical trauma (13) and can assess various surgical procedures in dogs (33, 117), peaking 24 h postoperatively (42). The short half-life of canine CRP (19 h) makes it a useful marker for identifying the intensity of mild clinical stressors (33, 118, 119) whose effects dissipate more rapidly.

It has been stated earlier that the slope of changes is a reliable predictor variable for the expected peak (13). A five-fold smoother slope in the elevation of CRP concentration generated a milder peak after instrument shank-assisted ovariohysterectomy, so its return level to the base value showed a significant reduction at T48 compared to that of the animals in the opposite group. Thus, it is suggested that future research on the slope of the post-OHE CRP increase would also be planned to reduce study duration and be used for designing and scheduling postoperative analgesia protocols and lengths. As CRP is elevated approximately 6 h after a single stimulation (119), the present data revealed that there is no clinical necessity for sampling before 6 h after surgery. Moreover, because serum concentration peaks between 24 and 48 h in dogs (42, 115, 119), and CRP has a short half-life (33, 118, 119), the last measurement time of 72 h after surgery was appropriate for CRP return.

It has been imagined that OVE can satisfy surgeons’ hopes by reducing the consequences of OHE. Moldal et al. did not find any differences in the levels of CRP, glucose, or iron in the blood between them. Therefore, the greatest trauma in OHE occurs during the surgeon’s manipulation of the ovarian pedicle (86). Thereby, the instrument shank-assisted method’s unique characteristic can be considered an advantage. By reducing manipulation of the ovarian pedicle through eliminating digital strumming, the surgical trauma has also been reduced to a level close to the lowest expected minimum. Experienced surgeons have the advantage of avoiding unnecessary organ manipulation (26, 27), which causes minimal surgical trauma and postoperative serum CRP concentration. Hence, the present modified technique, which entails less organ touch, presents surgical quality closer to that of an experienced surgeon.

It is not without merit to state that CRP concentrations seem to be a decent predictor of how invasive an operation is (120), despite some contradictory reports, such as that there is no correlation between the length of surgery and CRP, despite its rise (121), or that CRP concentrations may not be significant in the diagnosis of a disease (118). In the present study, the return to baseline after a moderate rise in CRP levels utilizing the instrument shank-assisted OHE, as contrasted to the conventional group’s high CRP levels, suggests that the instrument shank-assisted OHE could be concluded as having a minor invasive nature.

The risk of infection is increased when abnormal CRP responses are seen 5 or 7 days following surgery (116). A progressive decrease in serum CRP content in both groups could indicate the absence of infection. It is not unlikely that a procedure requiring fundamentally less organ manipulation would result in improved recovery, but ultimate healing was not the focus of this study.

Finally, the pattern of CRP changes followed the pain charts without correlation, according to the data. So, at least when the surgery is minor, there may not be a statistical correlation between changes in the CRP and pain. This may be because of the wide range of its reported changes. In this instance, the CRP profile may be able to anticipate pain patterns, but it cannot be used to make statistical conclusions.

The CRP changes showed that OVE and other similar procedures cannot be the final solution to animal comfort, and modification of the surgical technique on the more severe parts of the surgery is necessary, whether it is the method proposed in the current study or other solutions that may be introduced later. Although the graphs of CRP and pain changes appear similar, establishing a definitive relationship or statistically significant correlation between them requires further research.

## 5. Conclusion

The current study supported the practicability of a single-person ovariohysterectomy in deep-chested adult mixed-breed dogs without tearing the suspensory ligament, along with a reduction in surgical length, CRP, and pain. On the basis of the results, there are still questions regarding the efficiency of using serum CRP concentration as an alternative pain assessment indicator.

## 6. Limitations and future research

Future studies evaluating physiological values, pain, the amounts of analgesics and anesthetics consumed during the operation, hemorrhage, heat loss, instrument handling errors, wound size, and more tissue handling can help determine the surgical technique more precisely. Intraoperative evaluations need an up-to-date drug anesthesia protocol, which was not available in this study because certain nonsteroidal anti-inflammatory drugs were not available in the area and veterinarians were not allowed to use opioids or isoflurane. Further research will be needed to compare the performance of surgeons with varying degrees of expertise and animals of varying ages, making it more difficult to generalize the findings.

## Data availability statement

The original contributions presented in the study are included in the article/Supplementary material, further inquiries can be directed to the corresponding author.

## Ethics statement

The animal study was approved by Iranian biomedical research ethics committee IR.IAU.BABOL.REC.1399.004, IR.IAU.BABOL.REC.1399.015, and IR.IAU.BABOL.REC.1399.093. The study was conducted in accordance with the local legislation and institutional requirements.

## Author contributions

NZ: idea, project design, project implementation, statistical analysis, drawing illustrations manually, article writing, article translation, translation editing, and final proofing. SM: project design, project implementation, digitizing Illustrations, article writing, article translation, and translation editing. AB: project design, project implementation, article writing. NN: project design, project implementation, translation editing. SF: project implementation. All authors contributed to manuscript revision, read, and approved the submitted version.
